# Nef-M1, a peptide antagonist of CXCR4, inhibits tumor angiogenesis and epithelial-to-mesenchymal transition in colon and breast cancers

**DOI:** 10.18632/oncotarget.4615

**Published:** 2015-07-27

**Authors:** Venkat R. Katkoori, Marc D. Basson, Vincent C. Bond, Upender Manne, Harvey L. Bumpers

**Affiliations:** ^1^ Department of Surgery, Michigan State University, College of Human Medicine, Lansing, MI, USA; ^2^ Department of Microbiology, Immunology and Biochemistry, Morehouse School of Medicine, Atlanta, GA, USA; ^3^ Department of Pathology, University of Alabama at Birmingham, Birmingham, AL, USA

**Keywords:** CXCR4, Nef-M1 peptide, tumor angiogenesis, epithelial-to-mesenchymal transition, colorectal cancer, breast cancer

## Abstract

The Nef-M1 peptide competes effectively with the natural ligand of CXC chemokine receptor 4 (CXCR4), stromal cell-derived factor 1-alpha, to induce apoptosis and inhibit growth in colon cancer (CRC) and breast cancer (BC). Its role in tumor angiogenesis, and epithelial-to-mesenchymal transition (EMT) regulation, key steps involved in tumor growth and metastasis, are unknown. We evaluated the angioinhibitory effect of Nef-M1 peptide and examined its role in the inhibition of EMT in these cancers.

Colon (HT29) and breast (MDA-MB231) cancer cells expressing CXCR4 were studied *in vitro* and in xenograft tumors propagated in severe combined immunodeficient mice. The mice were treated intraperitoneally with Nef-M1 or scrambled amino acid sequence of Nef-M1 (sNef-M1) peptide, a negative control, starting at the time of tumor implantation. Sections from tumors were evaluated for tumor angiogenesis, as measured by microvessel density (MVD) based on immunostaining of endothelial markers. *In vitro* tumor angiogenesis was assessed by treating human umbilical vein endothelial cells with conditioned media from the tumor cell lines. A BC cell line (MDA-MB 468) which does not express CXCR4 was used to study the actions of Nef-M1 peptide. Western blot and immunofluorescence analyses assessed the effect of Nef-M1 on tumor angiogenesis and EMT in both tumors and cancer cells.

Metastatic lesions of CRC and BC expressed more CXCR4 than primary lesions. It was also found that tumors from mice treated with sNef-M1 had well established vascularity, while Nef-M1 treated tumors had very poor vascularization. Indeed, the mean MVD was lower in tumors from Nef-M1 treated mice than in sNef-M1 treated tumors. Nef-M1 treated tumor has poor morphology and loss of endothelial integrity. Although conditioned medium from CRC or BC cells supported HUVEC tube formation, the conditioned medium from Nef-M1 treated CRC or BC cells did not support tube formation. Western blot analyses revealed that Nef-M1 effectively suppressed the expression of VEGF-A in CRC and BC cells and tumors. This suggests that Nef-M1 treated CRC and BC cells are more consistent with E-cadherin signature, and thus appears more epithelial in nature.

Our data indicate that Nef-M1 peptide inhibits tumor angiogenesis and the oncogenic EMT process. Targeting the chemokine receptor, CXCR4, mediated pathways using Nef-M1 may prove to be a novel therapeutic approach for CRC and BC.

## INTRODUCTION

The American Cancer Society estimated that 136, 830 cases of colorectal cancer (CRC) and 235, 030 cases of invasive breast cancer (BC) will have occurred in 2014 [1]. CRC is the third most common cancer in American men and women and BC is the second most common cancer in women [1]. Few advances in the treatment of CRC and BC in the last half century have substantially reduced mortality from advanced disease. Therefore, identification of novel drugs that targets oncogenic molecular pathways and inhibits tumor progression is necessary to produce effective targeted treatments.

Previously, we demonstrated that an apoptotic peptide from the human immunodeficiency virus-1 (HIV-1) Nef protein, Nef-motif 1 (Nef-M1), was cytotoxic to various cultured human cancer cell lines, and we have characterized Nef- role in activation of apoptosis and inhibition of tumor growth of CRC or BC [2–4]. In the original studies, Nef, a 27–34 kD myristoylated protein expressed early in the infection cycle in host cells [5], was shown to compete with stromal cell-derived factor 1-alpha (SDF-1α) a natural ligand of CXC chemokine receptor 4 (CXCR4), and induce apoptosis during the infection [6].

Certain chemokines and their receptors, in particular SDF-1 α and CXCR4, are expressed in various epithelial cancer cells and favor cancer cell migration, proliferation, and survival [7–15]. CXCR4 expression was found to be independently associated with poor survival of CRC and BC patients [16, 17]. CXCR4 inhibition reduces tumor angiogenesis [18, 19] and in a mouse model, intraperitoneal CXCR4 inhibitors significantly reduced neovascularization in tumors [19].

Angiogenesis is necessary for both tumor growth and metastasis [20, 21]. Solid tumor growth requires the development of new blood vessels and there are correlations between vascularity, expression of proangiogenic factors, biologic aggressiveness, high pathologic grade, and poor survival [22]. Acquisition of a mesenchymal-like phenotype is associated with pro-metastatic properties, including elevation of mesenchymal markers, increased motility, invasion, drug resistance, immunosuppression, and cancer stem cell characteristics (20). Tumor angiogenesis and EMT are key targets for modern research in antitumor therapy. Most current therapies target the vascular endothelial growth factor (VEGF) and EMT pathways. However, not all tumors respond to inhibitors of VEGF or EMT, and some tumors that respond initially to VEGF blockers may become resistant during treatment. The angioinhibitory role of Nef-M1 peptide and underlying molecular mechanisms associated with Nef-M1/CXCR4 has not been established in CRC and BC. Elucidation of the function of CXCR4 and its signaling pathway during tumor progression will contribute to an understanding of tumorigenesis and metastasis. There is a need to explore new drugs targeting tumor angiogenesis and signaling pathways. In this report, the novelty is defining angioinhibitory effect of Nef-M1 peptide and to exploring inhibition of EMT process in CRC and BC that linked to Nef-M1 peptide /CXCR4 complex.

## RESULTS

### CXCR4 is expressed in tumor xenografts and parent human colon cancer cells

**Figure 1:**
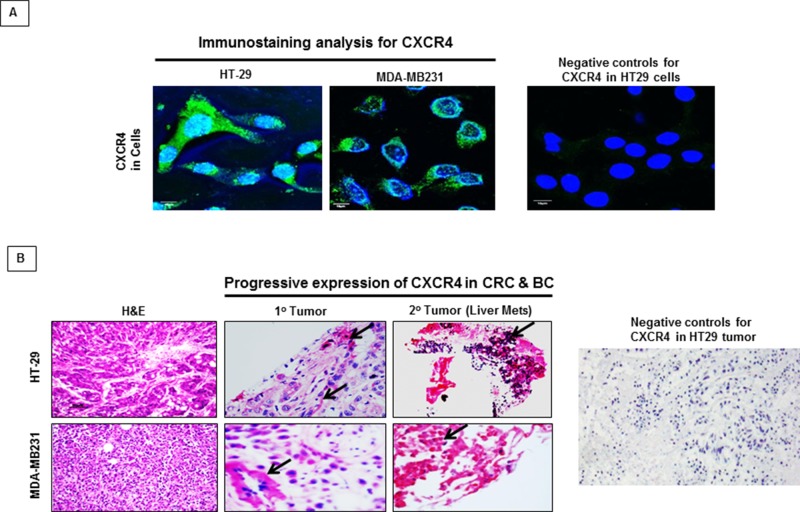
CXCR4 expression in CRC and BC by immunostaining **A.** Immunostaining confirmed the presence of CXCR4 expression in CRC (HT29) and BC (MDA-MB231). **B.** Compared to primary CRC and BC lesions, the expression of CXCR4 was markedly increased in metastatic CRC and BC lesions (red staining). Negative control without primary antibody for CXCR4 was used to show specificity.

We studied the expression of CXCR4 by immunostaining tumor xenografts and their parental human CRC and BC cell lines. Immunostaining revealed expression of CXCR4 in the xenografts as well as in the parental human CRC and BC cells growing *in vitro* (Figure 1A & 1B). H&E staining of the xenografts are included for tissue comparisons (Figure 1B). CXCR4 expression was observed in the nucleus, cell membrane and cytoplasm of CRC and BC. The expression of CXCR4 in lysates of tumor and parent cells was confirmed by western blot (data not shown).

### CXCR4 is progressively expressed in advanced disease

The expression of CXCR4 in paired primary and metastatic lesions of CRC and BC obtained from xenografts was compared. The expression of CXCR4 was substantially higher in metastatic tumors than in the corresponding primary tumors from the same animal (Figure 1B). This suggests that CXCR4 is progressively expressed as malignant disease becomes more advanced, thus there appears to be a direct association with tumor progression and metastasis.

### Nef-M1 peptide induces apoptosis

**Figure 2:**
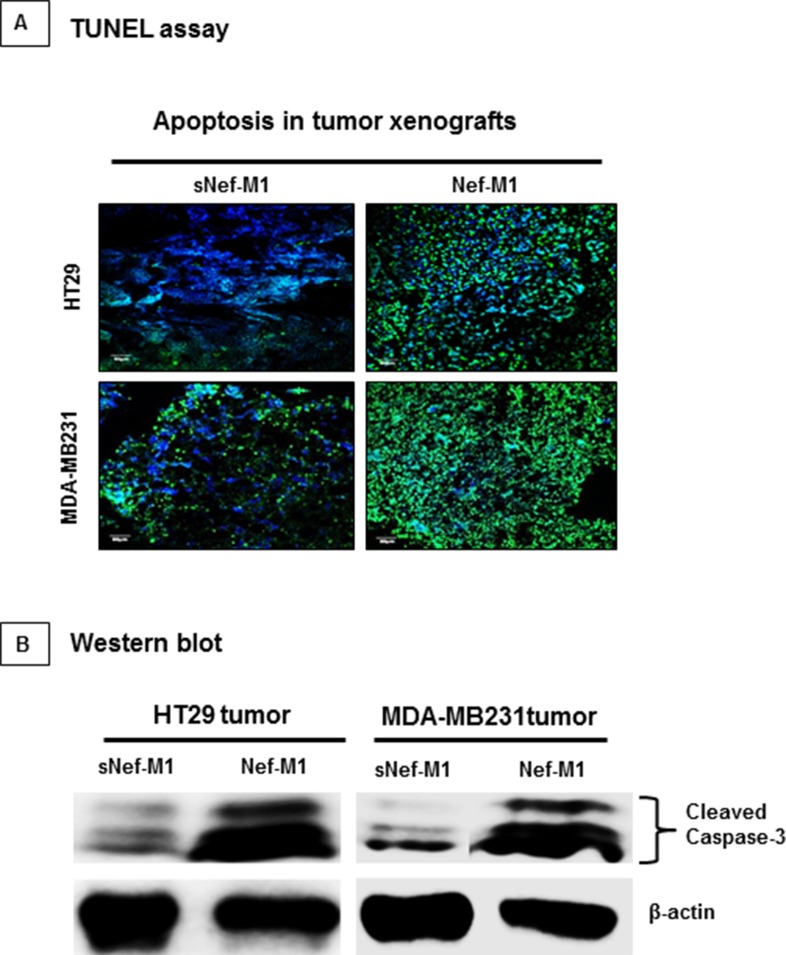
TUNEL assay on paraffin sections of representative CRC and BC, counterstained with DAPI **A.** The Nef-M1 peptide treated samples demonstrate increased nuclear fragmentation and fragment labeling at sites of DNA cleavage (intense green staining). **B.** Western blot analysis for caspase-3 activation on lysates of tumors. The Nef-M1 peptide induced apoptosis, as indicated by the presence of increased activated caspase-3 levels in Nef-M1 treated samples.

CXCR4 antagonist, Nef-M1 peptide activates apoptosis in CRC and BC cells *in vitro* [3, 4], but whether this occurs within intact tumors was not known. We evaluated the effects of Nef-M1 peptide on apoptosis in tumors of HT29 and MDA-MB231 by terminal deoxynucleotidyl transferase dUTP nick-end labeling (TUNEL) assay. Increased TUNEL labeling was observed (green punctuate labeling) at sites of DNA cleavage in the Nef-M1 treated tumors (Figure 2A). The percentage of TUNEL labeled nuclei in HT29 and MDA-MB231 tumors from mice treated with Nef-M1 peptide was 85% and 89.3% respectively. Nef-M1 induction of apoptosis was paralleled by the presence of more activated caspase-3 in tumors from Nef-M1 treated mice (Figure 2B) than from mice treated with sNef-M1.

### Nef-M1 peptide inhibits tumor angiogenesis

**Figure 3:**
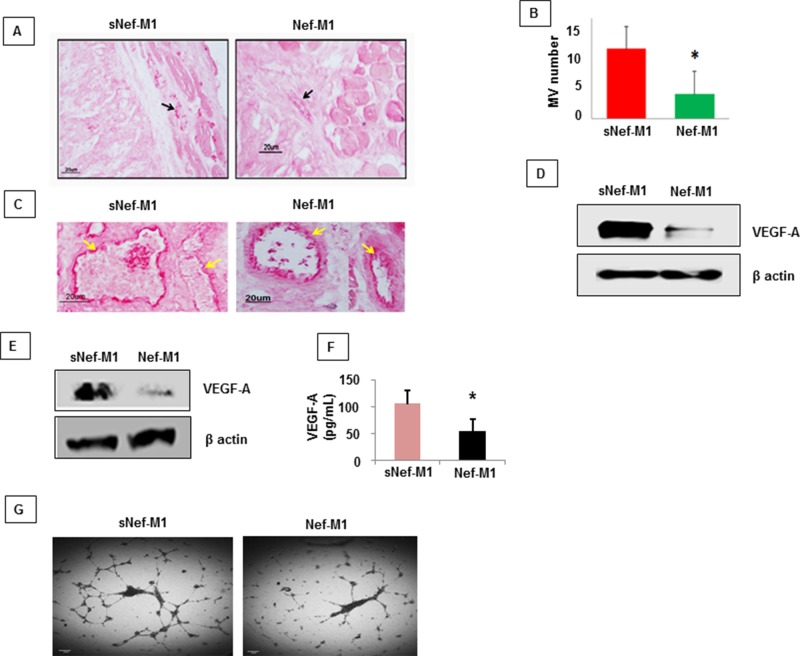
Effect of the Nef-M1 peptide on angiogenesis of CRC as determined by immunostaining for the endothelial marker CD31 **A.** Representative pictures of stained sections of CRC from mice treated with sNef-M1 or Nef-M1 peptide (x200 magnification). Microvessels were identified based on their morphology and highlighted by CD31-immunoreactive endothelial cells. **B.** Average microvessel density defined as the number of intratumoral vessels from fields in sections of CRC from mice treated with sNef-M1 or Nef-M1 peptide. Quantitation of microvessel density shows a significant inhibition of tumor angiogenesis by Nef-M1 peptide. **C.** Morphology associated with treatment. Nef-M1 treated tissue has poor morphology and loss of endothelial integrity. **D.** VEGF-A protein was assessed by western blot in lysate of tumors from mice treated with sNef-M1 or Nef-M1 peptide. Nef-M1 decreased expression of VEGF-A in CRC. **E.** VEGF-A protein was assessed by western blot in lysate of HT29 cells treated with sNef-M1 or Nef-M1 peptide. Nef-M1decreased expression of VEGF-A in HT29 cells. **F.** VEGF-A protein was assessed by ELISA in conditioned medium of HT29 cells treated with sNef-M1 or Nef-M1 peptide. The Nef-M1 peptide inhibited VEGF-A secretion. **G.** Effect of Nef-M1 peptide on tube formation by HUVECs. Nef-M1 inhibited capillary network formation.

**Figure 4:**
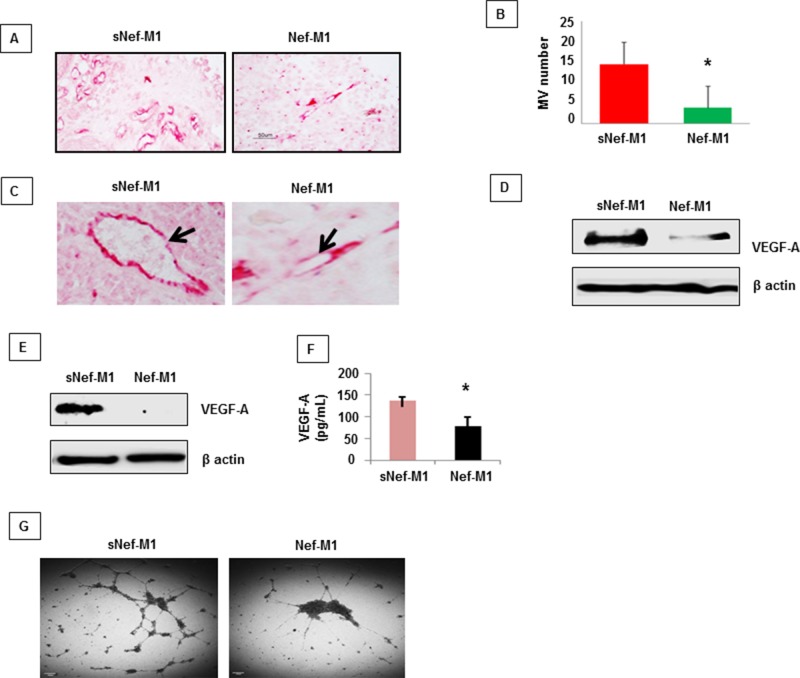
The effect of the Nef-M1 peptide on angiogenesis of BC as determined by immunostaining for CD31 **A.** Microvessels were identified based on their morphology in microvessels highlighted by CD31-immunoreactive endothelial cells. **B.** Nef-M1 significantly inhibited tumor angiogenesis. **C.** Nef-M1 disrupted vascular morphology. **D.** VEGF-A protein was assessed by western blot in lysate of tumors from mice treated with sNef-M1 or Nef-M1 peptide. Nef-M1 decreased expression of VEGF-A in BC. **E.** VEGF-A protein was assessed by western blot in lysate of MDA-MB231 cells treated with sNef-M1 or Nef-M1 peptide. Nef-M1 decreased expression of VEGF-A in MDA-MB231 cells. **F.** VEGF-A protein was assessed by ELISA in conditioned medium of MDA-MB231 cells treated with sNef-M1 or Nef-M1 peptide. The Nef-M1 peptide inhibited VEGF-A production. **G.** Effect of Nef-M1 peptide on tube formation by HUVECs. Nef-M1 inhibited capillary network formation.

The effect of the Nef-M1 peptide on tumor angiogenesis was initially evaluated by immunostaining for the endothelial marker CD31, a marker for well-established vascularity. Mice were treated with Nef-M1 or sNef-M1 peptide, starting at the time of tumor implantation. Immunostaining for CD31 indicated that control tumors (sNef-M1 peptide treated) had well established vascularity, but Nef-M1 peptide treated tumors had poor vascularization for both CRC (Figure 3) and BC (Figure 4). High expression of CD31in tumors is associated with a high degree of angiogenesis which implies rapid growth [22]. The average microvessel density (MVD) was decreased in Nef-M1 peptide treated tumors (*n* = 5) compared to sNef-M1 peptide treated tumors (*n* = 5) in CRC (Figure 3A & 3B). In BC, the average MVD was similarly decreased in Nef-M1 peptide treated tumors (*n* = 4) compared to sNef-M1 peptide control tumors (*n* = 5) (Figure 4A & 4B). Nef-M1 treated tissue has poor morphology and loss of endothelial integrity (Figure 3C & 4C) in both CRC and BC. Thus, control tumors (sNef-M1 peptide treated) maintained well established vascularity, while Nef-M1 peptide decreased the tumor vascularization significantly.

### Nef-M1 peptide decreases the expression of VEGF-A in CRC and BC

VEGF-A is a key proangiogenic factor released from cancer cells that stimulates vasculogenesis and angiogenesis [23]. In a tumor microenvironment, cancer cells secrete a high level of VEGF which binds to receptors on surrounding endothelial cells, promoting endothelial cell migration, proliferation, differentiation and tube formation [23]. Western blots of lysates of tumors from mice revealed significantly less VEGF-A protein in tumors from mice treated with Nef-M1 than in tumors from mice treated with sNef-M1 (Figure 3D & 4D). Similarly, western blot analyses of lysates of CRC and BC cells revealed that VEGF-A protein was significantly decreased in cells treated with Nef-M1 peptide compared to cells treated with sNef-M1 peptide control samples (Figure 3E & 4E). ELISA of VEGF secretion by CRC and BC cell lines revealed markedly decreased levels of VEGF in Nef-M1 treated CRC and BC cells (Figure 3F & 4F).

### Nef-M1 peptide inhibits endothelial cell tubular formation

HUVEC form tube-like structures on Matrigel, a vital first step in angiogenesis. We therefore next examined the formation of tubules by HUVEC in Matrigel to show a direct effect of the Nef-M1 peptide on endothelial cell function. HUVECs were plated at a density of 5 × 10^4^/well into individual wells of a 96-well cluster dish coated with Matrigel in the presence of conditioned medium collected from either Nef-M1 peptide or sNef-M1 peptide treated CRC or BC cells. The conditioned medium from sNef-M1 treated CRC and BC cells stimulated the HUVEC to form networks of multiple tube-like structures, while conditioned medium from Nef-M1 treated CRC and BC cells did not support tubule formation similarly to the conditioned medium. (Figure 3G & 4G).

### Nef-M1 peptide inhibits EMT process

**Figure 5:**
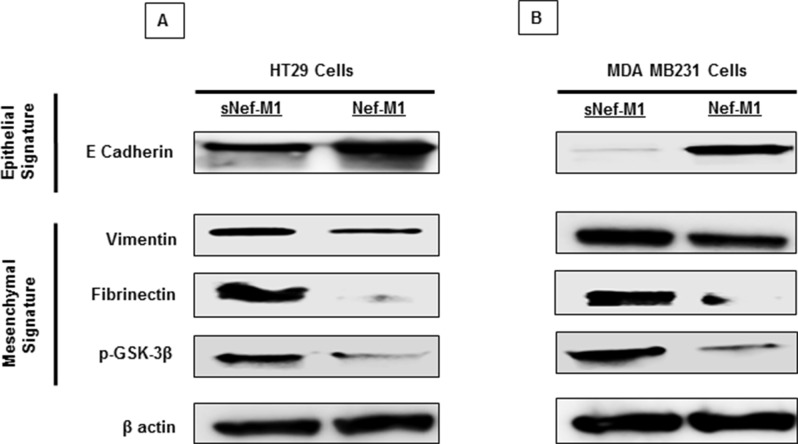
Nef-M1 peptide inhibits EMT in CRC and BC cells Western blot analysis showed increased expression of E-cadherin and decreased expression of vimentin, fibronectin and p-GSK-3β in CRC A. and BC B. cells treated with Nef-M1 peptide.

The effect of Nef-M1 peptide on the development of EMT related molecular signatures was evaluated. EMT is a phenotypic conversion that facilitates development of neoplasia, and is associated with tumor progression and metastasis [24]. During this process, E-cadherin (an epithelial marker) is down regulated and vimentin, fibrinectin, and p-GSK-3B (Mesenchymal markers) are upregulated. Nef-M1 treated tumor cells analyzed via western blot displayed increased expression of the epithelial signature E-cadherin and decreased expression of the mesenchymal signature vimentin, fibronectin, and p-GSK-3β (Figure 5A & 5B). Western blots of lysates of the mouse xenografts tumors also revealed significantly increased expression of E-cadherin protein and decreased expression of the mesenchymal signature proteins vimentin, fibronectin, and p-GSK-3β in tumors from the mice injected with Nef-M1 compared with tumors from sNef-M1treated control mice (data not shown). These results indicate that Nef-M1 may inhibit tumor progression through inhibiting the EMT process.

### Nef-M1 peptide inhibits tumor angiogenesis and EMT process in BC cells through CXCR4

**Figure 6:**
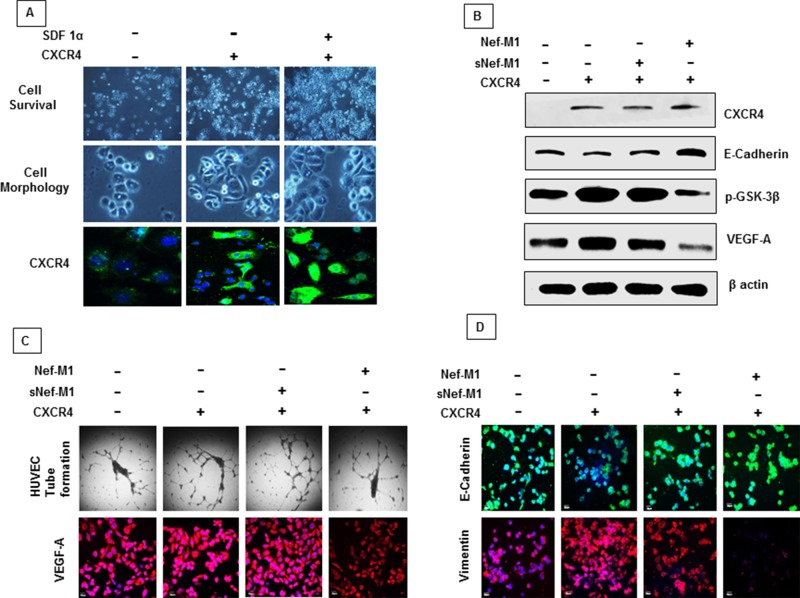
A. Proliferation and morphologic features of MDA-MB468 cells overexpressing CXCR4 Cell morphologic changes are shown in phase-contrast images. The CXCR4 and its ligand SDF-1α induces proliferation and morphological changes associated with tumor progression. SDF-1α induces internalization of CXCR4. **B.** Cell lysates from MDA-MB468 control or CXCR4-expressing cells were immunoblotted with antibodies for anti-CXCR4, E-cadherin, p-GSK-3β, VEGF-A or β-actin. Cells that express CXCR4 became susceptible to Nef-M1 peptide induced inhibition of p-GSK-3β expression (mesenchymal signature), VEGF-A expression (tumor angiogenesis) and induced elevation of E cadherin (epithelial signature). **C.** Cells that express CXCR4 became susceptible to Nef-M1 peptide induced inhibition of capillary network formation and VEGF-A expression as detected by tube formation assay and immunofluorescence respectively. **D.** Cells that express CXCR4 became susceptible to Nef-M1 peptide induced elevation of E cadherin (epithelial signature) and inhibition of vimentin expression (mesenchymal signature) as detected by immunofluorescence analysis.

CXCR4 signaling is frequently activated in human cancers [25]. CXCR4 has been found to be associated with several cellular pathways including proliferation and apoptosis [26–30]. Increased expression of this receptor and its ligand SDF-1α has been described in several malignancies [31]. CXCR4 expression is required for tumor initiation and progression [25]. Furthermore, we have previously shown that Nef-M1 peptide induced apoptosis through the CXCR4 receptor [6]. We reported that the breast tumor cell line MDA-MB468, which does not express CXCR4, is refractory to Nef-M1-induced apoptosis [6]. To further validate the relationship between CXCR4 expression, Nef-M1 sensitivity, and cell survival, we transiently transfected MDA-MB468 cells with a pCMV-CXCR4 expressing vector. The expression of CXCR4 in these cells was validated by immunofluorescence (green-cytoplasmic and cell membrane localization) and western blot (Figure 6A & 6B). A significant increase in cell survival was observed in CXCR4 transfected cells compared to survival in non-transfected cells. Survival was also increased in cells that express CXCR4 and in the presence of the CXCR4 ligand SDF-1α (Figure 6A). CXCR4 induced tube formation (Figure 6C) and morphological changes including cell polarization and extension of invadopodia associated with tumor progression (Figure 6A). SDF-1α induced internalization of CXCR4 in MDA-MB468 cells evidenced by confocal microscopy (Figure 6A). The expression of CXCR4 in BC cells was associated with increased expression of VEGF-A (Figure 6C), vimentin and p-GSK-3β and decreased expression of E cadherin (Figure 6B & 6D).

Given the potential roles of CXCR4 in tumorigenesis, MDA-MB468 cells were then used to study whether Nef-M1 peptide inhibits tumor angiogenesis and EMT through the CXCR4 receptor. We observed that the pCMV-CXCR4 transfected cells became susceptible to Nef-M1- induced inhibition of the development of a network of multiple tube-like structures as well as exhibiting reduced VEGF expression (angiogenesis) (Figure 6B & 6C). Furthermore, cells expressing CXCR4 became susceptible to Nef-M1-induced inhibition of p-GSK-3β and vimentin expression (mesenchymal signature) (Figure 6B & 6D) and Nef-M1 induced elevation of E cadherin (epithelial signature) (Figure 6B & 6D). This data strongly suggest that CXCR4 is a potential target of Nef-M1 peptide in the inhibition of tumor angiogenesis and oncogenic EMT process.

## DISCUSSION

We have previously shown that the Nef-M1 peptide is cytotoxic and inhibits the growth of CRC and BC [2–4]. Our present work demonstrates that the Nef-M1 peptide also inhibits tumor angiogenesis both *in vitro* and *in vivo*. Inhibition of tumor angiogenesis by Nef-M1 peptide correlates with decreased expression of VEGF-A. Moreover, Nef-M1 peptide also inhibits EMT.

Chemokine receptors, which belong to the family of G-protein-coupled receptors, are involved in regulation of the immune response, inflammation, leukocyte trafficking, and cytoskeletal rearrangement [32]. The chemokine receptor/ligand CXCR4/SDF-1α is unique in that SDF-1α is the only known ligand for this receptor [33–36]. CXCR4 is the most common chemokine receptor in solid human cancers, including breast [37], melanoma [8], renal cell [38], brain [9, 39], thyroid [10, 40], non-small cell lung [11, 41], pancreatic [12, 42], ovarian [13, 43], prostate [14, 44], and colorectal [15, 45] cancers. Activation of molecular pathways such as p38 mitogen-activated protein kinase (p38 MAPK) induces tumor progression and is associated with CXCR4 expression [26–28]. CXCR4 expression is higher in embryonic or dedifferentiated cells than in normal cells [46], and CXCR4 expression independently predicted poor survival in tumors [47]. Consistent with previous studies, this study showed that there was substantially increased expression of CXCR4 in metastatic lesions of tumors compared to the primary lesions of tumors in both CRC and BC. It is important to explore new drugs that target CXCR4 or interrupt SDF-1α/CXCR4 complex which could have a profound impact as a therapeutic agent to suppress tumor progression.

Agents specifically directed against the CXCR4 receptor have been developed [48, 49]. By blocking the receptor from interacting with its natural ligand, inhibition of primary tumor growth and metastasis can be achieved [50]. These synthetic CXCR4 antagonists, originally created to combat HIV-1, do not eliminate cells, but rather compete with the SDF-1α ligand to inhibit cellular function. It is important to note a distinct difference between these agents and Nef-M1 peptide. The Nef-M1 peptide induces apoptosis in tumor cells, thus eliminating the cell. Nef-M1 efficiently activates caspase-3, a key molecule of the apoptotic process, *in vivo*. Indeed, the high percentage of TUNEL-labeled nuclei in tumors from mice treated with Nef-M1 peptide demonstrates the parallel induction of apoptosis by this peptide. Interestingly, a recent study reported that SIVmac239-Nef decreases cell surface expression of the CXCR4 in COS-7 cells and decreases proliferation, and migration of tumor cells [51]. However, SIVmac239-Nef has no effect on caspase 3 activation and there are no sequence similarities between Nef-M1 peptide and SIVmac239-Nef, although both are subsets of the full Nef protein. Furthermore, since the SIVmac239-Nef does not induce apoptosis, it does not eliminate the cancer cells as Nef-M1 does. Our prior and current studies suggest that the Nef-M1 peptide is a potential therapeutic agent that can be used to target CXCR4 for induction of apoptosis in CRC and BC.

Tumor implantation, growth, and metastasis are correlated with neovascularization and angiogenesis [52, 53]. Tumor vascularization is possible through sprouting angiogenesis from preexisting vessels or through recruitment of circulating endothelial cells or progenitors, which may contribute to different extents depending on the molecular context [54–56]. Endothelial cells are stimulated by tumor-released growth factors to migrate and divide at the tumor site, ultimately forming blood vessel tubes stabilized by smooth muscle cells [57].

Angiogenic factors act through many signaling pathways. The VEGF pathway and Notch signaling are two of the most important mechanisms associated with embryonic vascular development and tumor angiogenesis [58, 59]. Increased expression of CXCR4 induces tumor metastasis through enhanced proliferation of cells by activating molecular pathways [29] and through accelerating vascularization by activating VEGF [17]. CXCR4 signaling [30], which increases VEGF-A promoter activity [60] can promote angiogenesis and thus enhance tumor viability. We demonstrate here that the expression of CXCR4 in BC cells is associated with increased expression of VEGF-A. Increased expression of VEGF and its receptors correlates with increased MVD, cell proliferation, and tumor growth rate, which impairs patient survival in diverse cancers [23, 61, 62]. Although we focused on VEGF here, there are many other pro-angiogenic growth factors such as fibroblast growth factors (FGFs) [63], placental growth factor (PIGF) [64], and platelet-derived growth factor (PDGF) [65]. Each of these pro-angiogenic growth factors also regulates angiogenesis through CXCR4 mediated signaling [66]. Therefore, CXCR4 is an attractive target since it is the most common chemokine receptor expressed in cancer cells and correlates factors related to angiogenesis. Thus, targeting of CXCR4 by an appropriate therapeutic agent may be a means of controlling the tumor progression. We have shown the inhibitory role of Nef-M1 on tumor angiogenesis and expression of VEGF-A. This study also demonstrated correlation between decreased expression VEGF-A and inhibition of tumor angiogenesis in both tumors and corresponding parent cells, suggesting Nef-M1 peptide may inhibits tumor angiogenesis by inhibiting CXCR4/VEGF signaling mechanism in CRC and BC.

EMT, the change from an epithelial phenotype into a mesenchymal phenotype, is an important characteristic of cancer stem cells [67]. CXCR4 is a key regulator of the EMT process through which it could activate signals associated with tumor progression [68]. Blockade of SDF-1/CXCR4 signaling inhibits expression of MMP-9 and vimentin [69], invasion-related phenotypes known to promote metastatic signaling. The present study demonstrated that Nef-M1 targets CXCR4, inhibits EMT, and inhibits tumor progression.

VEGF expression has been found to be more pronounced in CXCR4 expressing cancer cells [70]. The present study demonstrated a significant association between CXCR4 and cell survival. Because expression of CXCR4 in BC cells has been found to be associated with increased tube formation (angiogenesis), and induction of EMT, we used a BC cell line that does not express CXCR4 to study whether Nef-M1 peptide inhibits angiogenesis or inhibits EMT process through the CXCR4 receptor. Indeed, the CXCR4 expressing cells became vulnerable to Nef-M1 peptide induced inhibition of tube formation and reduced VEGF expression (angiogenesis). Furthermore, cells that express CXCR4 became susceptible to the shift from a more mesenchymal to a more epithelial profile in response to Nef-M1. This data strongly suggest that CXCR4 is a potential target of Nef-M1 peptide in the inhibition of tumor angiogenesis and oncogenic EMT process in both CRC and BC.

Although additional studies will be warranted to elucidate the molecular mechanisms that contribute to the angioinhibitory activity of Nef-M1 peptide, our results suggest that Nef-M1 peptide could be a potential drug lead compound for therapeutic applications for the treatment of cancers.

## MATERIALS AND METHODS

### Peptides and antibodies

Nef-M1 and sNef-M1 peptides were obtained from CPC Scientific Inc (Sunnyvale, CA). Antibodies used in this study were purchased from Cell Signaling Technology Inc (Danvers, MA), Novus Biologicals (Littleton CO), Santa Cruz Biotechnology Inc (Dallas, TX), Sigma-Aldrich (St. Louis, MO) and Abcam (Cambridge, MA). Information on primary antibodies has been provided in Table 1.

**Table 1:** Details of primary antibody used in this study

S.No	Antibody	Catalog#	Host	Clonality	Company	Dilutions for IHC/IF	Dilutions for WB
1	CXCR4	NB600-786	Rabbit	Polyclonal	Novus Biologicals,	1:500	1:1000
2	β-actin	4967S	Rabbit	Polyclonal	Cell Signaling Technology	-	1:1000
3	VEGF-A	Sc-507	Rabbit	Polyclonal	Santa Cruz Biotechnology	1:500	1:1000
4	CD31	Ab28364	Rabbit	Polyclonal	abcam	1:200	-
5	E-cadherin	Sc-7870	Rabbit	Polyclonal	Santa Cruz Biotechnology	1:200	1:1000
6	Vimentin	3932S	Rabbit	Polyclonal	Cell Signaling Technology	1:500	1:1000
7	Fibrinectin	F3648	Rabbit	Polyclonal	Sigma-Aldrich	-	1:1000
8	Phospho-GSK3-3β	9323P	Rabbit	Monoclonal	Cell Signaling Technology	-	1:1000
9	Cleaved-caspase-3	9664p	Rabbit	Monoclonal	Cell Signaling Technology	-	1:1000

IHC; Immunohistochemistry, IF; Immunofluorescence, WB; Western Blot

### Animals and Nef-M1 peptide injections

#### Animals and tumor growth

Severe Combined Immunodeficiency (SCID) mice were obtained from Taconic Farm (Taconic, NY) at approximately one month of age. After one week of quarantine, mice were inoculated with colon (HT29) and breast cancer (MDA-MB-231) cells (1 × 10^6^ cells/0.1 cc) subcutaneously using Hanks balanced salt solution to establish primary tumors. The animals received standard rodent chow and water, and resided in isolated micro-filtered cages in rooms designated for immune compromised mice. The mice were checked daily to assess health status and tumor growth. Body weight, nutritional intake, general activity level, and ruffling of mice fur served as our indicators of health status. Surgical procedures were done using disposable gowns, sterile gloves, and a laminar flow hood. Gloved hands were wet with a liquid sterilant before making direct contact with the mice.

### Surgical procedures

All surgical procedures were conducted under the guidelines and approvals of IACUC.

### Tumor implantation

For tissue implants, after developing a solid tumor following cell injection, the solid tumor was cut into 2–4 mm pieces in serum-free culture media and kept at 4°C until used. The mice were sedated using 0.6 mL of avertin (2, 2, 2-tri-bromoethanol and 2-methyl-2-butanol). Tumors were implanted into the subQ. All surgical wound closures were made using a 5–0 absorbable suture or skin staples. Following implantation the mice were placed under heat lamp for 10 min to recover and then placed back in their cages. Approximately two hours post procedure we again check for full recovery and stability.

### Nef M1 treatment

SCID mice were injected intraperitoneally at one week post tumor implantation with either the active Nef M1peptide or the scrambled amino sequence of Nef-M1peptide (sNef-M1). Each treatment group represents at least 5 mice. Nef peptide dilutions were made in phosphate buffer saline (PBS) at a concentration of 2 μg per 0.1 mL as optimized and Nef-M1 peptide injections were done biweekly for four weeks for all treatment groups as described in our previous reports. For *in vitro* cell culture studies, dilutions and concentrations of the Nef-M1 peptide or sNef-M1 peptide (100 ng/mL) were accomplished according to a previously reported protocol [2–4]. Briefly, dose responses were assessed by incubating 2.5 × 10^5^ cancer cells with the Nef-M1 peptide or sNef-M1peptide at various concentrations in 35-mm multiwall plates for 24 hr. The concentrations of Nef-M1 peptide were 0, 0.01, 0.1, 1, 10, and 100 ng/mL. Nef-M1 peptide dose response was determined by terminal deoxynucleotidyl transferase dUTP nick-end labeling (4).

### Metastasis model

SCID mouse model was used to establish the hepatic metastatic lesion as reported in our earlier version (2). Hepatic metastasis was produced injecting HT29 and MBA-MD231 cells, a line derived from a primary carcinoma of the colon and breast, into the spleen with the cell count (1 × 10^6^ cells/0.1 cc). Briefly, splenic injections were done by having the spleens extracorporeally injected under direct vision and then replacing the spleen in its usual anatomical location. Livers were removed from mice at necropsy. The number of gross lesions in the liver was counted by use of a magnification lens. These lesions and corresponding primary tumors were used to assess progressive expression status of CXCR4.

### TUNEL assay

To evaluate apoptosis, TUNEL assays were performed with an in situ cell death detection kit (EMD Millipore, Billerica MA). The procedure for immunohistochemical detection and quantification of apoptosis was based on labeling of DNA breaks. Deparaffinization for tissue sections was performed with xylene followed by carrying rehydration procedure through a series of ethanol solutions (100%, 95%, 80% and 50%). Tissue sections were rinsed with 1xPBS and incubated in permeabilization solution (0.1% Triton X-100, 0.1 sodium citrate) for 10 min at room temperature. The sections were rinsed with 1xPBS, and 50 μL of TUNEL reaction mixture, consisting of TdT and biotinylated nucleotides was added. The cells were incubated in a humidified chamber for 1 h at 37°C and rinsed three times with 1xPBS. Confocal images were acquired using the Olympus FluoView FV1000 Confocal Laser Scanning Microscope (Olympus America, Inc., Center Valley, PA) configured on a fully automated inverted Ix81microscope using a 40x UPLFLN oil (NA1.3) objective. Western blot analysis of caspase-3 activation in lysates of CRC and BC samples was performed. Caspase-3 activation was determined by Western blot analysis identifying cleavage of the 32 kDa pro-caspase-3 protein into two smaller 17 kDa and 12 kDa caspase-3 proteins.

### Cell cultures, transfections and treatment

One CRC cell line (HT29), two BC (MDA-MB231 and MDA-MB468) cell line and one human umbilical vein endothelial cell (HUVEC) line were used. Each cell line was originally purchased from American Type Culture Collection (ATCC, Manassas, VA) and cryopreserved. CRC and BC cell lines were cultured in 5% CO_2_ at 37°C in RPMI 1640 medium (Life Technologies, Grand Island, NY) supplemented with L-glutamine, 10% fetal bovine serum (Life Technologies), and penicillin (100 U/mL)/streptomycin (100 U/mL) (Life Technologies). HUVEC cells were cultured in vascular cell basal medium (ATCC) supplemented with growth supplements of endothelial cell growth kit-VEGF (ATCC) and penicillin (100 U/mL)/streptomycin (100 U/mL). Dilutions and concentrations of the Nef-M1 peptide or sNef-M1 peptide (100 ng/mL) were accomplished according to a previously reported protocol [2–4]. Cell cultures were grown to 80% confluence and treated with either sNef-M1 or Nef-M1 peptide according to an established protocol.

A BC cell line (MDA-MB 468) that does not express CXCR4 was used to study whether Nef-M1 peptide acts through the CXCR4 receptor. These cells were transiently transfected with pCMV control vector or pCMV-CXCR4 expressing vector using TurboFect transfection reagent (Fisher Thermo Scientific, Waltham, MA) for the indicated times according to the manufacturer's instructions. Briefly, 1 × 10^5^ cells were seeded into six-well plates containing medium and incubated overnight. For each well, 4 μg DNA (pCMV or pCMV-CXCR4) was mixed with 100 μL of RPMI-1640. The mixture was then combined with a solution of 2 μL of TurboFect transfection reagent. After a 20-min incubation period at room temperature, the mixture was applied to the cells in final volume of 2 ml. After 24 h, cells were treated with 100 ng/mL of peptides (sNef-M1, Nef-M1 or SDF-1α). Then, the cells were cultured for an additional 24 h in 5% CO_2_ at 37°C before analysis.

### Histological staining and immunocytochemistry

Immunohistochemistry was performed as previously described [71, 72]. Paraffin blocks for tumors of parent cells were prepared with standard histopathology methods. Briefly, tumors were collected, washed twice in 1xPBS, and fixed in formalin free Zinc fixative for 30-min, then washed again. These structures were subsequently embedded in paraffin blocks and sectioned at 5 micron thickness. Deparaffinization of tissue sections was performed with xylene followed by rehydration through a series of ethanol solutions (100%, 95%, 80% and 50%). Sections from tumors were evaluated for CXCR4 expression and tumor angiogenesis, as measured by MVD based on immunostaining of endothelial marker (CD31). MVD was determined by light microscopy in areas of invasive tumor containing the highest numbers of microvessels per area. Individual microvessel counts were made on a 200x field within the areas of most intense tumor neovascularization.

CRC and BC cells were prepared by plating cells (1 × 10^5^) on glass slides with poly-D-lysine (Becton Dickinson, Franklin Lakes, NJ) and then allowing cells to attach overnight. Cells were subsequently washed with 1xPBS and fixed with formalin free Zinc fixative (Becton Dickinson) for 30-min and washed again. Cells and deparaffinized tissue sections were then permeabilized with 1xPBS containing 0.1% Triton X-100 for 5-min and washed with 1xPBS. For immunofluorescence analysis, blocking was performed for cells, and deparaffinized tissue sections with 200 μL of serum blocking solution (Zyagen, San Diego, CA) followed by incubation at room temperature for 60-min in a humidified chamber. The slides of the samples were incubated with 200 μL (2 μg/mL) of rabbit polyclonal anti-human specific antibody for overnight at 4°C at room temperature. Samples were washed 3 times with 1xPBS for 5-min each then incubated with 200 μL of biotinylated secondary antibody solution (Zyagen) for 30 min at room temperature. After washing with 1xPBS, samples were covered with 200 μL of streptavidin-FITC conjugate solution (Zyagen) and incubated for 30-min at room temperature. Samples were washed 3 times again with 1xPBS, then counterstained with 4′, 6-Diamidino-2-Phenylindole, Dilactate (DAPI) solution (Zyagen) for 2-min. Samples were washed 3 times with 1xPBS for 5-min each and covered with coverslip on to slides using anti-fade fluorescent mounting medium (Zyagen). Confocal images were acquired using the Olympus FluoView FV1000 Confocal Laser Scanning Microscope (Olympus America) configured on a fully automated inverted Ix81microscope using a 40x UPLFLN oil (NA1.3) objective. Negative control without primary antibody for CXCR4 was used to show its specificity.

### Enzyme-linked immunosorbant assay

VEGF-A protein that had been released into the conditioned medium of HT29 and MDA-MB231 cells was measured using a commercially available human VEGF ELISA Kit (Life Technologies). Cells (5 × 10^5^) were seeded in six-well plates in 2 mL of complete growth medium. Twenty-four hours later, cells were serum-starved for 24 h and then exposed to Nef-M1 peptide with RPM1 1640 containing 2% FBS. After 24 h of incubation in 5% CO_2_ at 37°C and 95% humidified air to allow VEGF protein secretion, the conditioned medium (CM) was collected. The supernatant was clarified by centrifugation for 5-min, aliquoted, and stored at −70°C until analysis. CM was concentrated by Amicon Ultra Centrifugal Filter Devices (EMD Millipore). VEGF-A levels in culture supernatants were assayed using a quantitative human VEGF ELISA Kit (Life Technologies) according to the manufacturer's instructions. In brief, CM (50 μL) was incubated with 50 μL of assay diluents for 2 h at room temperature in a 96-well tissue culture plate coated with a mAb against VEGF-A. After four washes, 100 μL of biotinylated Hu VEGF conjugate was added into each well, and the mixture was incubated for 1 h at room temperature. Following the subsequent addition of 100 μL streptavidin-HRP working solution to each well and incubation of 30-min at room temperature. After washing, 100 μL of stabilized chromagen was added to each well and incubated for 30-min. The absorbance was measured at 450 nm using an Infinite^®^ M1000 PRO microplate reader (Tecon US, Inc. Morrisville, NC). For standardization, serial dilutions of recombinant human VEGF-A were assayed simultaneously. All experiments were performed in triplicate.

### Tube formation assay

After Matrigel (EMD Millipore) was thawed on ice; 96-well plates coated with 50 μL Matrigel in each well were incubated at 37°C for 30-min to allow the Matrigel to polymerize. To examine the effect of Nef-M1 peptide on tumor cell-induced tube formation of HUVECs, a conditioned medium was collected from Nef-M1 or sNef-M1 peptide treated HT29, MDA-MB231 cells and also from MDA-MB468 cells as indicated and used as the growth medium for HUVECs. A total of 1 × 10^4^ HUVECs were seeded into each well that had conditioned medium. Cells were then incubated for 8 h to allow formation of tube-like structures. Endothelial cell tube formation was assessed with an inverted photomicroscope. Tubular structures were quantified by manually counting the number of capillaries in low-power fields.

### Western blot analysis

CRC and BC cells or tissues were prepared for Western blot by incubating with lysis solution (1.0% Nonidet P-40; 50 mM Tris-HCl; pH 7.5; 20 mM EDTA buffer) (Sigma-Aldrich, St. Louis, MO) at room temperature for 5-min. The lysates were centrifuged for 20-min at 12, 000 rpm at 4°C. The supernatants were collected and stored at −70°C. Protein concentrations were determined with the Bradford assay kit (Bio-Rad Laboratories, Hercules, CA). Portions of each sample (20 μl) were separated by SDS-PAGE on a 4–20% Tris-HCl Criterion precast gel (Bio-Rad Laboratories) and electrophoretically transferred to polyvinylidene difluoride (PVDF) membranes (Life Technologies). The membranes were washed in 1x Tris-buffered saline (TBS) for 5-min, and then blocked with 5% nonfat milk in 1x TTBS (1x TBS and 0.1% Tween 20) for 1 h by shaking at room temperature. For detection of expression status of protein in lysates, a rabbit polyclonal anti-human specific antibody was used. This was accomplished by shaking the membranes at 4°C overnight, as directed by the manufacturer, followed by application of horseradish peroxidase (HRP)-conjugated goat anti-rabbit antibody. Protein bands were detected by SuperSignal West Femto Maximum Sensitivity Substrate Reagent (Fisher Scientific, Pittsburgh PA), followed by exposure on an Odyssey Fc Dual-Mode Imaging System (LI-COR, Lincoln, Nebraska). Images were scanned into Adobe Photoshop 5.0.2, and densitometry was performed using Scion Imaging software, Release Beta 3b (Scion Corporation, Frederick, MD). After detection of specific proteins, the blots were stripped and hybridized with a polyclonal rabbit anti-β-actin, then probed with the HRP-conjugated anti-rabbit antibody for normalization.

### Statistical analysis

Statistical analysis was performed using the standard Pearson's X2-test, Student's two-tailed *t*-test, Fisher's exact test, or one-way ANOVA for comparisons. Differences were deemed statistically significant at *p* ≤ 0.05.
